# Worldwide Estimation of Parental Acceptance of COVID-19 Vaccine for Their Children: A Systematic Review and Meta-Analysis

**DOI:** 10.3390/vaccines11030533

**Published:** 2023-02-24

**Authors:** Zainab Alimoradi, Chung-Ying Lin, Amir H. Pakpour

**Affiliations:** 1 Social Determinants of Health Research Center, Research Institute for Prevention of Non-Communicable Diseases, Qazvin University of Medical Sciences, Qazvin 3415613911, Iran; 2 Institute of Allied Health Sciences, College of Medicine, National Cheng Kung University, Tainan 701401, Taiwan; 3 Biostatistics Consulting Center, National Cheng Kung University Hospital, College of Medicine, National Cheng Kung University, Tainan 701401, Taiwan; 4 Department of Public Health, College of Medicine, National Cheng Kung University, Tainan 701401, Taiwan; 5 Department of Occupational Therapy, College of Medicine, National Cheng Kung University, Tainan 701401, Taiwan; 6 Department of Nursing, School of Health and Welfare, Jönköping University, SE-551 11 Jönköping, Sweden

**Keywords:** child, COVID-19, vaccine acceptance, vaccine hesitancy

## Abstract

Currently, the best method to well control the spread of COVID-19 without severe mental health problems is to reach herd immunity. Therefore, the vaccination rate of the COVID-19 vaccine is critical. Among the populations, children are the vulnerable ones to get vaccinated; therefore, it is important to assess parents’ and guardians’ willingness to have their children vaccinated. The present systematic review and meta-analysis synthesized evidence to estimate the parents’ acceptance rate of COVID-19 vaccination toward their children. Additionally, factors explaining the acceptance rate were investigated. Four academic databases (PubMed, Scopus, Web of Science, and ProQuest) together with Google Scholar were searched, and the references of the included publications were searched as well. Using the PECO-S framework (population, exposure, comparison, outcome, and study design), observational studies of cross-sectional, cohort, or case-control studies were included. The outcome was parents’ or guardians’ willingness to let their children be vaccinated. The studies included in the present review were restricted to English and peer-reviewed papers published between December 2019 and July 2022. A total of 98 papers across 69 different countries with 413,590 participants were included. The mean age of the parents was 39.10 (range: 18–70) years and that of their children was 8.45 (range: 0–18) years. The pooled estimated prevalence of parental acceptance to vaccinate their children with the COVID-19 vaccine was 57% (98 studies, 95% CI: 52–62%, I^2^: 99.92%, τ^2^: 0.06). Moreover, data collection time was a significant factor explaining parental willingness in the multivariable meta-regression, with a 13% decrease in parental willingness by each month increase in time, explaining 11.44% of variance. Qualitative synthesis results showed that parents’ COVID-19 vaccine knowledge, trust in theCOVID-19 vaccine, and facilitators in vaccination (e.g., low cost, good vaccine accessibility, and government incentive) were significant factors for higher willingness, while mental health problems (e.g., having worries and psychological distress) were significant factors for lower willingness. Given that the acceptance rate was relatively low (57%) and does not achieve the requirement of herd immunity (i.e., 70%), governments and healthcare authorities should try to elevate parents’ knowledge and trust in the COVID-19 vaccine, facilitate in vaccination, and reduce their mental difficulties to improve the overall vaccination rate among children.

## 1. Introduction

On 11 March 2020, WHO declared COVID-19 as a global pandemic, which has led to serious disruptions in the economy and society [1,2,3,4,5,6]. Based on the WHO COVID-19 dashboard, as of 31 January 2023, there have been 753,479,439 confirmed cases of COVID-19, including 6,812,798 deaths, globally [7]. Although the control of human movement, including travel restrictions and quarantine, is an effective containment and mitigation strategy for COVID-19, it can lead to psychological problems and a significant social and economic burden [8,9]. Lack of motivation to follow recommendations, resulting from long-term public health measures and restrictions, may contribute to the resurgence of COVID-19 cases [10]. Various physical measures such as wearing masks and social distancing have been implemented to contain the spread of this virus. For children, reducing time in school (e.g., campus closure with online learning at home) has been the primary approach [11]. However, social isolation has negative effects on children’s mental health [12].

In order to overcome the limitations of school time reduction during the pandemic, immunization seems to be the safest and most cost-effective health intervention implemented throughout history, saving millions of lives annually [13,14]. To date, immunization programs against several infectious diseases have been successfully implemented worldwide and have been able to control diseases such as smallpox, polio, diphtheria, pertussis, and rubella [15]. Vaccination is the key to reducing the incidence of COVID-19, which enables children to continue their daily activities [16]. Due to the emergence of some new types of highly transmissible COVID-19 strains, different parties (including the scientists, healthcare providers, and governments) acknowledge the importance of high vaccination uptake for herd immunity [17,18,19], which would reduce the effect of the vaccine on transmission, pathogenicity, and hospitalization rates associated with COVID-19 [20,21]. Therefore, willingness to be vaccinated plays a key role in government vaccination calls to slow down the spread of the virus [22]. However, vaccination hesitancy, which according to the World Health Organization (WHO) poses a threat to global health, has become a significant issue during the COVID-19 pandemic [23,24,25]. In a recent systematic review of 31 peer-reviewed papers, different acceptance rates of COVID-19 vaccination among the general population were reported from 23.6% to 97% across different countries [26,27]. In another narrative review, data regarding COVID-19 vaccine acceptance rate were collected from surveys in 114 countries/territories. Acceptance rates ≥60% were seen in 72/114 countries/territories, with 42 countries/territories having rates between 13% and 59% [28]. Additionally, different acceptance rates at a national level were reported in one recent systematic review in the US: 12 to 91.4% acceptance rate [29]. Factors such as educational qualification, compliance with preventive measures related to COVID-19, age, gender, source of information related to COVID-19, history of influenza vaccination, inefficient government efforts and initiatives, and trust in the government are among the factors affecting the enthusiasm for vaccination against COVID-19 [27,30,31,32,33,34].

Vaccination in children often requires the consent of their parents or guardians [35]. To facilitate the implementation of the COVID-19 vaccine among children, it is thus important to understand parental acceptance of their children’s COVID-19 vaccination and the associated barriers and facilitators. However, current research suggests that parental compliance and influencing factors vary considerably across studies. For example, a systematic review of 44 studies involving 317,055 parents found that the overall proportion of parents planning to vaccinate their children against COVID-19 was 60.1% with heterogeneity ranging from 25.6 to 92.2% [36]. Similar variations were reported in another systematic review including 29 studies from 16 countries and regions with 68,327 participants [37], showing that vaccination willingness could be as high as 91.4% [38] or as low as 21.6% [39].

The purpose of this study is to investigate the prevalence and factors affecting the acceptance of the COVID-19 vaccine for children under legal age among their parents. Specifically, the present study investigated children’s vaccination attitudes, whether their parents have been vaccinated against COVID-19, parents’ age, etc. According to our literature review, prior systematic review and meta-analysis studies did not consider the diverse subgroups when synthesizing data to examine the variables related to the acceptance of the COVID-19 vaccine, especially for children. Moreover, the latest studies recommend new vaccines against COVID-19 for children and adolescents [40,41], which results in difficult decision making for parents and caregivers to vaccinate their children. For this reason, the aim of the current research was to draw a comprehensive and related picture of various factors and attitudes related to this decision. The information obtained will help to provide a better understanding for further research as well as for health authorities and professionals to respond to potential problems in an adequate and targeted manner.

### Study Aims

This study aimed primarily to estimate the prevalence of parental acceptance/willingness to vaccinate their children with the COVID-19 vaccine. The secondary aims were:

- Assessment of heterogeneity and its possible sources for estimated pooled prevalence of parental acceptance/willingness to vaccinate their children with the COVID-19 vaccine;
- Moderator analyses to determine influential variables sources for estimated pooled prevalence of parental acceptance/willingness to vaccinate their children with the COVID-19 vaccine;
- Determining influential factors for parents to accept COVID-19 vaccination for their children.

## 2. Materials and Methods

### 2.1. Protocol and Registration

The study protocol was registered in the PROSPERO, International prospective register of systematic reviews under decree code of CRD42022333337 [42]. The findings of this systematic review are reported based on the Preferred Reporting Items for Systematic Reviews and Meta-Analyses (PRISMA) reporting guideline [43].

### 2.2. Systematic Review Questions

The systematic review question was formulated using PECO-S framework. The PECO-S is a framework to formulate search questions assessing associations between exposures and outcomes in various fields of health [44]. The PECO components are P for Population, E for Exposure, C for Comparison, O for Outcome, and S for Study design. The present study was designed to answer the following main research question:

What is the worldwide estimated prevalence of parental acceptance to vaccinate their children with the COVID-19 vaccine?

### 2.3. Eligibility Criteria

The eligibility criteria based on PECO components were set as follows: (1) Population: parents or children’s guardian with no limitation regarding their demographic characteristics; (2) Exposure: COVID-19 pandemic; (3) Comparison: other populations other than children; (4) Outcome: Frequency or prevalence of COVID-19 vaccination acceptance (and/or no hesitance) or willingness to receive COVID-19 vaccines for children; and (5) Study design: observational studies including cross sectional, cohort, or case-control design.

Other eligibility criteria include being published between December 2019 and July 2022, using English language, published as a peer-reviewed paper, reporting data on frequency or prevalence of parents or children’s guardian acceptance for their children’s COVID-19 vaccination.

### 2.4. Information Sources

Academic databases including PubMed, Scopus, Web of Science (WoS), and ProQuest were systematically searched from the beginning of December 2019 to the end of July 2022. To have a more comprehensive search, reference lists of the included publications and medRxiv were independently searched.

### 2.5. Search Strategy

The main search terms included COVID-19, vaccine, parents, and children. The search strategy was developed using Boolean operators (AND, OR). The advanced search attributes of each database were considered and customizing the search syntax was adapted.

### 2.6. Study Selection

In the first step, the title and abstract of all retrieved papers during the electronic and manual search processes were evaluated based on the inclusion criteria. This was followed by examination of the full texts of the potentially relevant articles based on the above-mentioned criteria. These processes were performed independently by two reviewers. Initial disagreements about the selection of studies were resolved through discussions.

### 2.7. Data Collection Process and Data Items

Data were extracted and recorded in pre-designed Excel datasheets by two reviewers independently. The following data were abstracted from each study: first-author name; country in which the study had been conducted as well as its income level and development status based on World Bank data; sample size; data collection date; parents and children ages; country location based on WHO regions; type of study; quality of study; and raw data to calculate prevalence of parents’ willingness of their children to be vaccinated against COVID-19.

### 2.8. Risk of Bias in Individual Studies

The Newcastle–Ottawa Scale (NOS) was used to assess risk of bias within included studies. This checklist evaluates the methodological quality of observational studies in the following three sections: selection, comparability, and outcome [45,46]. The maximal acquirable score on the NOS checklist is 9 for each study. Studies with less than five points were classified as having a high risk of bias [45]. Methodological quality status was not considered as an eligibility criterion. However, the effect of methodological quality on the pooled effect size was assessed in the subgroup analysis and meta-regression.

### 2.9. Summary Measures and Data Synthesis

The selected summary measure of the present study for meta-analysis was the frequency or prevalence of the acceptance of the COVID-19 vaccine and their 95% confidence intervals (CIs). Numerical evidence regarding the prevalence of the COVID-19 vaccine acceptance was quantitatively synthesized using STATA software version 14. Meta-analysis using a random-effects model was conducted to consider both within-study and between-study variances [47]. Severity of heterogeneity was estimated using the I^2^ index [48].

Contributing factors influencing acceptance of COVID-19 vaccines were pooled using a meta-synthesis approach due to methodological heterogeneity of variables and measures.

### 2.10. Risk of Bias across Studies

Funnel plot and Begg’s test were used to assess publication bias [49]. Meta-trim with the fill and trim method was used to correct probable publication bias [50]. The Jackknife method was used for sensitivity analysis and probable single study effect on pooled effect size [51].

### 2.11. Additional Analyses

To investigate moderators for COVID-19 vaccine acceptance, subgroup analysis and meta-regression were conducted.

## 3. Results

### 3.1. Study Screening and Selection Process

**Figure 1:**
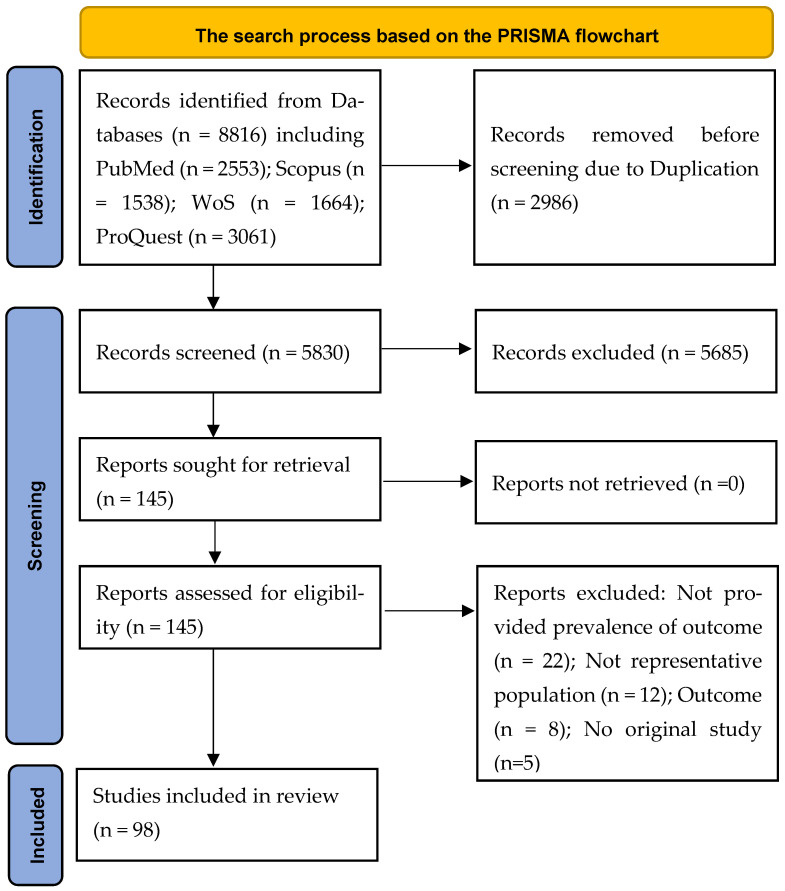
The search process based on the PRISMA flowchart. A total of 98 studies were finally included in the present systematic review and meta-analysis.

The initial search in four academic databases as well as Google Scholar resulted in retrieval of 8816 records: PubMed (n = 2553); Scopus (n = 1538); WoS (n = 1664); and ProQuest (n = 3061). After removing duplicates (n = 2986), the remaining manuscripts were screened based on their titles and abstracts. Finally, 145 papers appeared to be potentially eligible and their full texts were reviewed. In this process, 98 studies were pooled in the meta-analysis. The search process based on the PRISMA flowchart is illustrated in Figure 1.

### 3.2. Description of the Included Studies

Ninety-eight studies comprised 413,590 participants from 69 different countries (Albania, Aruba, Argentina, Antigua and Barbuda, Australia, Bahamas, Bangladesh, Belize, Bolivia, Brazil, Barbados, Canada, China, Chile, Colombia, Costa Rica, Cuba, Cayman Islands, Dominica, Dominican Republic, Ecuador, El Salvador, England, Germany, Greece, Grenada, Guatemala, Guyana, Haiti, Honduras, Hong Kong, India, Iraq, Israel, Italy, Jamaica, Japan, Jordan, Korea, México, New Zealand, Nicaragua, Panama, Paraguay, Peru, Philippines, Poland, Puerto Rico, Qatar, Russia, Saint Maarten, Saudi Arabia, Singapore, South Africa, Spain, St. Kitts and Nevis, St. Lucia, St. Vincent and the Grenadines, Switzerland, Suriname, Taiwan, Trinidad and Tobago, Turkey, Turks and Caicos Isl., UK, USA, Uruguay, Venezuela, Virgin Islands) were included. The highest number of studies were, respectively, conducted in AMR region (Americas; 27 studies), EUR region (Europe; 24 studies), WPR region (Western Pacific; 23 studies), EMR region (Eastern Mediterranean; 17 studies) and SEAR (South-East Asia; 5 studies). Three studies were conducted as multi-country studies. Half of the studies (49 out of 98) were conducted in developed countries with high income (61 out of 98). The smallest sample size was 50 (from the U.S.), and the largest sample size was 227,740 (from Latin America and the Caribbean). Most study respondents (68.1%) were mothers. The mean age of parents was 39.10 (range between 18 and 70 years) and that of their children was 8.45 years (range between 0 and 18 years). Almost all studies used a cross-sectional design, with only two studies adopting a longitudinal design. The first study was conducted during February 2020 in China and the last one was conducted during January 2022 in Saudi Arabia. Table 1 provides the summary characteristics of all included studies.

### 3.3. Methodological Quality Appraisal

**Figure 2:**
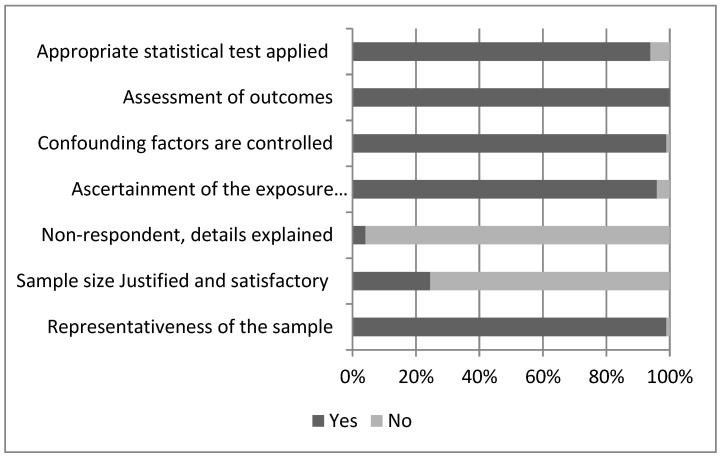
Details of the methodological quality appraisal of the included studies. In total, 58 of 98 studies were categorized as being low quality.

Most studies (58 out of 98) were categorized as being low-quality (or high risk of bias) studies. The total score of methodological quality is provided in (Table 1), with details in (Figure 2). The main methodological problems were: (1) description of the response rate or the characteristics of the responders and the non-responders not having been reported (94 out of 98 studies); (2) explanation regarding sample size estimation and justification not having been reported (74 out of 98 studies).

### 3.4. Pooled Prevalence of COVID-19 Vaccine Willingness

**Figure 3:**
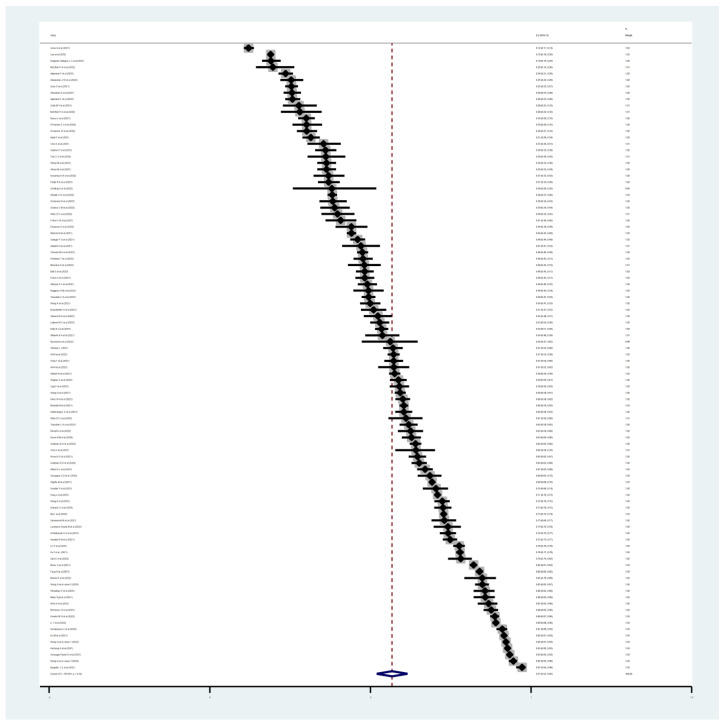
The forest plot of pooled prevalence of parental acceptance to vaccinate their children with the COVID-19 vaccine. The pooled estimated prevalence of parental acceptance to vaccinate their children with the COVID-19 vaccine was 57%.

The pooled estimated prevalence of parental acceptance to vaccinate their children with COVID-19 vaccine was 57% (98 studies, 95% CI: 52–62%, I^2^: 99.92%, τ^2^: 0.06). Figure 3 provides the forest plot regarding the pooled prevalence of parental acceptance to vaccinate their children with the COVID-19 vaccine.

**Figure 4:**
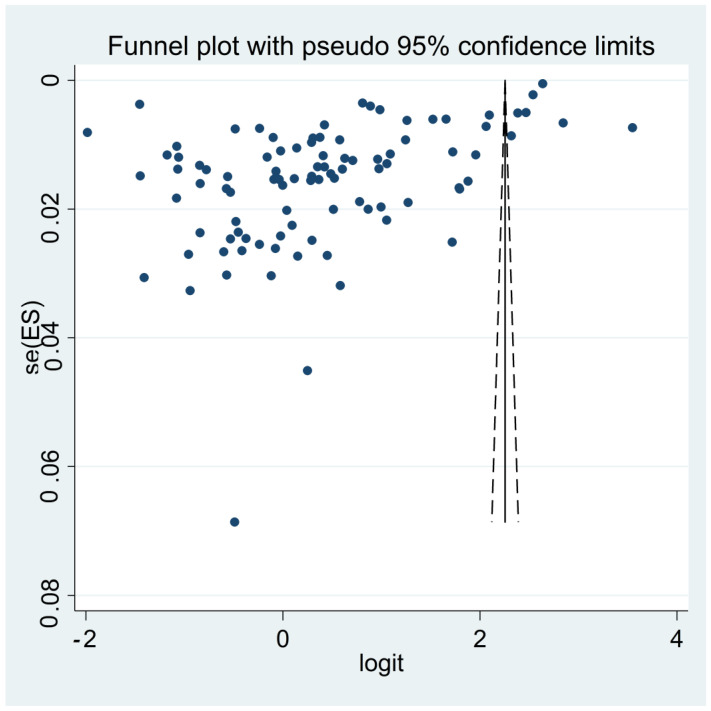
The funnel plot assessing publication bias among included studies reporting pooled prevalence of parental acceptance to vaccinate their children with COVID-19 vaccine. Publication bias seems probable.

The probability of publication bias was assessed using Begg’s test (*p* < 0.001) and funnel plot. Based on the asymmetric funnel plot (Figure 4), publication bias seems probable. The fill-and-trim method was used to correct probable publication bias, but no study was imputed, and publication bias was ruled out. Sensitivity analysis (based on the one-out or Jack-knife method) showed that the pooled effect size was not affected by a single-study effect.

### 3.5. Predictor Variables of Parental Willingness

Predictors of COVID-19 vaccine willingness were assessed using subgroup analysis (Table 2) and univariable and multivariable meta-regression (Table 3 and Table 4). Country income level, country location in WHO’s regions (i.e., AMR, EUR, WPR, EMR, and SEAR), and data collection method were significant moderators (*p* = 0.01) of parents’ willingness to vaccinate their children with the COVID-19 vaccine. High-income countries had the lowest prevalence of parental willingness (52%) compared to low- and upper-intermediate-income countries (62 and 65%, respectively). The lowest prevalence of parental willingness was observed in countries located in EMR compared to other regions (45% in EMR vs. 58% in AMR, 62% SEAR, 52% EUR, and 67% WPR). Data collection method was another significant variable influencing the pooled estimated parental willingness (*p* = 0.02). Studies collected data using phone interview had the lowest prevalence of willingness (35%). In the uni-variable meta-regression, data collection time was the only significant variable (*p* = 0.001) in predicting parental willingness. Multivariable meta-regression revealed that both data collection time (13% decrease in willingness by each increase in month) and country income level (7% decrease by increasing level of country income) were only significant predictors of parental willingness, which explained 11.44% of variance. None of the examined variables affect the heterogeneity.

### 3.6. Contributing Factors of Parental Willingness

Two main categories of contributing factors (i.e., family-related factors and vaccine-related factors) were identified among included studies using a qualitative synthesized approach.

#### 3.6.1. Family Related Contributing Factors

Parent self-vaccination [54,64,90,92,101,113,117,119,120,122,125,127,128,132,134,136] or their willingness for self-vaccination [65,76,77,86,88,92,98,103,125,127,129] showed positive association with parents’ willingness to vaccinate their children in almost all studies. Just one study reported that among participants vaccinated against COVID-19, only 29.0% were willing to vaccinate their children [80].

History of COVID infection in parents, children, or family members did not have consistent results among included studies, showing positive association [113,120,127,135], negative association [100,116], or no significant difference [87,88].

Parents’ age showed associations with their willingness to vaccinate their children. In most studies [38,52,58,62,65,74,86,87,98,110,113,116,119,123,124,125,131,133,135,139], parents with older age showed more willingness, while some inconsistent results were reported regarding higher vaccination hesitation among older parents [75,79,80].

Mothers showed more hesitancy regarding their children’s vaccination compared to fathers in almost all studies [52,57,68,75,79,91,92,93,98,106,116,133]; however, mothers also had higher willingness than fathers to vaccinate their children [83,88,105,115,120], although a few studies found no difference between mothers and fathers [87,130].

Source of information regarding the vaccine influenced parents’ decision for their children’s vaccination. When they received information from healthcare providers, physicians, or pediatrics, they reported more willingness to vaccinate their children [59,80,84,96,99,128,134,136]. Social media played different roles. In most studies, participants reported that social media and exposure to negative information increased parent hesitancy regarding vaccination [75,93,94,105,106], while some others reported a positive influence of social media for increasing parent acceptance [66,70].

Parents reported more willingness to vaccinate children if their children are older [57,75,90,99,100,108,111,118,119,124,125,134,135,138,139].

Parents with a higher number of children reported inconsistent decisions regarding willingness to accept their children’s vaccination. A higher number of children was associated with less willingness in some studies [38,55,87,105,108], higher willingness in other studies [99,102], or no difference in one study [88].

Parents’ higher levels of stress, anxiety, and psychological distress were associated with less intention for children’s vaccination [53,78,89,113]. Only one study reported that higher vaccine acceptability was associated with higher levels of anxiety regarding COVID-19 infection [142].

Parents’ education level showed inconsistent results in association with parental willingness for children’s vaccination. Some studies showed positive association of parents’ higher level of education with vaccination willingness [38,54,58,59,62,75,76,79,87,89,90,91,93,98,110,126,134], while others reported negative [52,74,84,113,116,119,137] or null [88] association.

Parents’ higher economic status showed positively higher association with parents’ willingness to vaccinate their children in almost all included studies [38,54,55,59,77,80,87,88,89,93,95,96,97,98,111,113,125], with only two studies reporting lower vaccination intention among lower income parents [52,137].

Parents having chronic conditions reported higher prevalence of vaccination willingness for their children in three studies [83,113], while lower willingness [57] and no difference [87] each was reported in one study. Additionally, parents whose children have a history of adverse vaccine reactions and allergies were less willing to vaccinate their children [52].

Parents living in rural and sub-urban areas showed less willingness compared to those in urban areas [105,113,125], except for one study reporting higher willingness of rural parents [52].

#### 3.6.2. Vaccine Related Factors

Parents reported more children’s vaccination willingness when they believe that children vaccination is necessary to halt the pandemic and to reach a better national economic situation [65,70,72,77,83,86,97,103,115,120].

Worry about vaccine safety and its potential adverse effect [56,59,64,65,67,70,72,76,77,81,85,86,90,91,92,94,95,98,101,103,105,112,115,116,118,121,126,127,128,131,132,133,140,142], novelty of vaccine and its’ short development time [57,101,116], and hesitancy regarding vaccine’s efficacy and benefits [56,59,66,70,71,72,74,76,92,94,101,105,115,117,118,122,126,128,131,132,133] were among the main predictors of vaccination hesitation of parents. When they perceived more worry about the vaccine’s adverse effect and hesitation regarding its’ efficacy, they preferred not to vaccinate their children.

Cost of vaccine [56,59,73], vaccine accessibility [59,63,101,123,141], and governmental incentive of giving a green pass after vaccination [101,123] were among of contributing factors for parents’ decision-making for their willingness to vaccinate their children.

Trust in the COVID-19 vaccine [59,98,121,126,128,142], trust in governments [104,121], and trust to health system [60,63,72,80,89] were also contributing factors.

Parents who reported more positive attitudes/beliefs toward vaccination [65,83,86,87,107,109] and who had more knowledge on vaccines (vaccine literacy) [58,61,65,79,83,86,88,89,97,108,109,110,121,139,142] had more willingness to vaccinate their children.

Obtaining influenza immunization was a positive contributing factor for parents to accept their children’s COVID-19 vaccination [57,59,65,68,77,86,104,116,121,140]. 

Parents who perceived the risk of COVID-19 transition from children to adults [60,106,118] and who perceived risk of children’s infection and being hospitalized because of COVID-19 [59,92,99,106,115,118,121,127,128,134] reported more willingness to vaccinate their children.

Comparisons between domestic and foreign vaccine preference have been investigated in very few studies, and domestic vaccines were preferred by parents for themselves and their children [56,142].

## 4. Discussion

In order to provide thorough estimation regarding parents’ willingness to have their children vaccinated, the present systematic review and meta-analysis synthesized data from studies published between December 2019 and July 2022 (98 papers). Apart from using meta-analysis to quantitatively synthesize parents’ acceptance rate of having children vaccinated, quantitative (i.e., meta-regression and subgroup analysis) and qualitative approaches have been used to synthesize the factors explaining parents’ willingness on their children’s vaccination. The synthesized results showed that the pooled estimated rate of parents’ willingness was 57% (95% CI = 52–62%). The low willingness to let children get vaccinated concurs with prior results reported by meta-analysis: 60.1% [32]. Although the present meta-analysis also revealed high heterogeneity (I^2^ > 90%) as similar to previous meta-analyses [32,33], the 95% CI in the present meta-analysis was narrower (52–62% vs. 25.6–92.2% and 21.6–91.4%). Nevertheless, the parents generally had low willingness to let their children get vaccinated. Consequently, it is important to know the potential reasons increasing or decreasing parents’ willingness to let their children get vaccinated. Apart from the quantitative finding, qualitative synthesis in the present review showed that the positive factors on parents’ willingness to vaccinate their children were knowledge on the COVID-19 vaccine, trust in the COVID-19 vaccine, and facilitators in vaccination (e.g., low cost, good vaccine accessibility, and government incentive); negative factors were parents’ mental difficulties, including worries, anxiety, and psychological distress.

Although having similar point-estimation in the parents’ willingness to have their children vaccinated, the present systematic review and meta-analysis had a narrower 95% CI than the two previous systematic review and meta-analyses [36,37]. A potential reason is that the papers included in their meta-analyses [36,37] were fewer than the present one, which resulted in an unstable estimation in the 95% CI. Specifically, Galanis et al. reviewed 44 papers [36] and Chen et al. reviewed 29 papers [37], while the present systematic review and meta-analysis reviewed 98 papers. With a double-size reviewed papers, the present systematic review and meta-analysis is likely to have a more precise estimation in the acceptance rate, especially in the interval-estimation.

Data collection time was a significant factor explaining dropped willingness (13% decreased by each month increased). This can be explained by risk compensation [143,144] and diffusion of responsibility with bystander effect [145,146]. It seems that when time progresses and the percent of vaccinated people increases, parents may feel safe not to let their children get COVID-19 vaccinated as other people have already been vaccinated. This point was pointed in regression analysis based on percent of vaccinated people in countries’ national level, which showed that each increased percent in percent of vaccinated people contributes to a 1% decrease in parental willingness. In other words, parents feel that the risk of COVID-19 infection for their children is decreased and they would not like to let their children vaccinated as a type of risk compensation [143,144]. Additionally, when the vaccination rate increases, parents may feel their responsibility of letting their children get vaccinated decreased, which is a phenomenon of diffusion of responsibility and bystander effect [145,146].

Although the parents’ willingness to have their children vaccinated, it is important to maintain the vaccination rate across time to adhere to the herd immunity. That is, a decreased vaccination rate may cause another wave of COVID-19 outbreak as documented in the literature. Therefore, governments and health authorities should have appropriate methods to maintain willingness of having children vaccinated among parents. The present systematic review and meta-analysis used the qualitative synthesis to find the importance of parents’ COVID-19 vaccine knowledge, trust in COVID-19 vaccine, and facilitators in vaccination (e.g., low cost, good vaccine accessibility, and government incentive) to improve their willingness to have children vaccinated. Moreover, mental health problems (e.g., having worries and with high levels of psychological distress) might reduce parents’ willingness to have their children vaccinated. Therefore, government and healthcare authorities should consider building campaigns on COVID-19 vaccine knowledge improvement, COVID-19 vaccine support systems, and psychological distress reduction to elevate the parents’ willingness to have their children vaccinated.

### Strengths and Limitations

The strengths of the present systematic review and meta-analysis included (i) a comprehensive search of the literature across WHO-defined regions (i.e., included AMR, EUR, WPR, EMR, and SEAR) to cover different ethnic and country populations; (ii) the use of robust methodology in reviewing papers (i.e., using the NOS checklist to evaluate each paper’s methodological quality; applying subgroup analysis and meta-regression to examine the moderator effects on parents’ willingness of having children vaccinated); (iii) synthesized findings from both quantitative and qualitative approaches to identify all potential factors explaining parents’ willingness of having children vaccinated.

There are some limitations in the present systematic review and meta-analysis. First, the COVID-19 pandemic had different severities and progresses across the studied papers because different countries controlled the COVID-19 pandemic with different situations. Therefore, it is hard to control this important confounder when cumulating the empirical data across countries worldwide. Most of the studies did not report data regarding the time window between vaccines availability at national level or time of vaccine approval for different age groups and collecting the data. Second, almost all papers analyzed in the present systematic review and meta-analysis did not use a standardized instrument assessing willingness to children’s vaccination. Subsequently, there might have been some measurement biases across the analyzed papers. Third, over half of the papers were at high risk of bias and the findings of the present systematic review and meta-analysis could be affected by the high risk of bias. Therefore, additional studies with low risk of bias are needed to further investigate the issue regarding parents’ willingness to have their children vaccinated. Fourth, although the present systematic review and meta-analysis had sought several academic databases (e.g., PubMed) and Google Scholar, the Google’s search engine was not used to search for potential grey literature. Therefore, it is possible that some relevant articles might not be included in the present systematic review and meta-analysis. Lastly, the following important information was not able to retrieve from the analyzed studies in the present systematic review and confounded the present findings: definitions of children (and the actual age ranges used for the analyzed studies); available vaccine (brands or types); and the vaccination procedure (e.g., whether the vaccines have been politicized in other countries as they have been in the US).

## 5. Conclusions

In conclusion, the present systematic review and meta-analysis updates the current understanding of parents’ willingness and hesitancy of letting their children get vaccinated. The willingness of the parents was generally low (mean acceptance rate: 57%; 95% CI: 52–62%), although it was highly heterogeneous (I^2^ = 99.92%). Moreover, time appeared to be the primarily significant factor explaining high levels of acceptance. Qualitative synthesized results showed that parents’ knowledge on COVID-19 vaccine, trust in COVID-19 vaccine, and facilitators in vaccination (e.g., low cost, good vaccine accessibility, and government incentive) could improve parents’ acceptance of children vaccination. In contrast, parents’ mental difficulties (e.g., having worries and psychological distress) were barriers to improve their willingness.

## Figures and Tables

**Table 1:** Summarized characteristics of included studies.

First Author, Publication Year	Data Collection Time	Country, WHO Region, Developing Status, Income Level	N Mother s % Parents Mean Age Children Mid-Range of Age	Study Design Sampling Method Recruitment Method	NOS Score/ Category
Yang, J. et al., 2021 [52]	2020.02	China, WPR, Developing, Upper intermediate income	12,872 73.09 34 NR	cross sectional Non-random sampling online	5/ High risk of bias
Yılmazbaş, P. et al., 2020 [53]	2020.04	USA, AMR, Developed, High income	440 70.5 33.5 5	cross sectional Non-random sampling online	4/ High risk of bias
Kelly, B.J. et al., 2021 [54]	2020.04	USA, AMR, Developed, High income	2247 52 41.5 9	cross sectional Non-random sampling online	5/ High risk of bias
Bell, S. et al., 2020 [55]	2020.04 to 05	England, EUR, Developed, High income	1252 95 42 1	cross sectional Non-random sampling online	5/ High risk of bias
Lin, Y. et al., 2021 [56]	2020.05	China, WPR, Developing, Upper intermediate income	2026 48.5 34 6.5	cross sectional Non-random sampling online	5/ High risk of bias
Goldman, R.D. et al., 2020 [57]	2020.04 to 05	six countries, Developed	1541 NR 39.9 7.5	cross sectional Non-random sampling online	5/ High risk of bias
Goldman, R.D. et al., 2022 [58]	2020.03 to 06	USA, AMR, Developed, High income	2687 75.36 35 9.5	cohort Not identified online	5/ High risk of bias
Hetherington, E. et al., 2021 [59]	2020.05 to 06	Canada, AMR, Developed, High income	1321 100 42.2 10.5	cohort Not identified self-administration	6/ Low risk of bias
Ennaceur, S. et al., 2022 [60]	2020.03 to 06	Saudi Arabia, EMR, Developing, High income	379 49.6 29 NR	cross sectional Non-random sampling online	5/ High risk of bias
Brandstetter, S. et al., 2021 [61]	2020.05	Germany, EUR, Developed, High income	612 NR NR 3.4	cross sectional Non-random sampling online	5/ High risk of bias
Davis, M.M. et al., 2020 [62]	2020.06	USA, AMR, Developed, High income	1008 55 41.5 NR	cross sectional Non-random sampling self-administration	5/ High risk of bias
Gjini, E. et al., 2022 [63]	2020.06	Albania, EUR, Developing, Upper intermediate income	475 89.6 41.5 4	cross sectional Non-random sampling self-administration	5/ High risk of bias
Kezhong, A. et al., 2021 [64]	2020.06 to 07	China, WPR, Developing, Upper intermediate income	13,451 NR 34.5 9	cross sectional Non-random sampling online	6/ Low risk of bias
Çelik, M.Y. et al., 2021 [65]	2020.07 to 09	Turkey, EUR, Developed, Upper intermediate income	274 65.7 36 3.5	cross sectional Non-random sampling online	5/ High risk of bias
Zhang, K.C. et al., 2020 [66]	2020.09	China, WPR, Developing, Upper intermediate income	1052 62.5 41.5 9.5	cross sectional Non-random sampling online	6/ Low risk of bias
Wang, Q. et al- wave 1, 2022 [67]	2020.09 to 10	China, WPR, Developing, Upper intermediate income	2881 74.5 41.5 NR	cross sectional Non-random sampling online	7/ Low risk of bias
Letterie, M.C. et al., 2022 [68]	2020.10 to 11	Korea, SEAR, Developed, High income	1066 58 41.5 NR	cross sectional Random sampling online	5/ High risk of bias
Wang, Q. et al., 2021 [69]	2020.09 to 10	China, WPR, Developing, Upper intermediate income	3079 49.2 45.5 9	cross sectional Non-random sampling self-administration	7/ Low risk of bias
Wang, Z. et al., 2021 [70]	2020.10 to 11	China, WPR, Developing, Upper intermediate income	1332 89.4 41.5 9.5	cross sectional Non-random sampling online	6/ Low risk of bias
Altulahi, N. et al., 2021 [71]	2020.11 to 12	Saudi Arabia, EMR, Developing, High income	3038 54.2 34 9	cross sectional Non-random sampling online	6/ Low risk of bias
Skjefte, M. et al., 2021 [72]	2020.11	Sixteen countries	17,054 100 41.5 NR	cross sectional Non-random sampling online	5/ High risk of bias
Reindl, D. et al., 2022 [73]	2020.11	USA, AMR, Developed, High income	582 56 41.5 NR	cross sectional Non-random sampling online	5/ High risk of bias
Feng, H. et al., 2021 [74]	2020.11 to 2021.01	China, WPR, Developing, Upper intermediate income	3703 57.1 40 NR	cross sectional Non-random sampling face to face interview	5/ High risk of bias
Montalti, M. et al., 2021 [75]	2020.12 to 2021.01	Italy, EUR, Developed, High income	4993 76.56 41.5 9	cross sectional Non-random sampling online	5/ High risk of bias
Padhi, B.K. et al., 2022 [76]	2020.11 to 2021.01	China, WPR, Developing, Upper intermediate income	770 39.6 41.5 9	cross sectional Non-random sampling online	6/ Low risk of bias
Humble, R.M. et al., 2021 [77]	2020.12	Canada, AMR, Developed, High income	1435 55.3 41 8.5	cross sectional Non-random sampling online	6/ Low risk of bias
Xu, Y. et al., 2021 [78]	2020.12	China, WPR, Developing, Upper intermediate income	4430 76 41.5 9.5	cross sectional Non-random sampling online	6/ Low risk of bias
Du, M. et al., 2021 [79]	2020.12 to 2021.01	China, WPR, Developing, Upper intermediate income	3011 100 41.5 9.5	cross sectional Random sampling online	7/ Low risk of bias
Alsulaiman, J.W. et al., 2022 [80]	2021.10 to 11	Jordan, EMR, Developing, Upper intermediate income	564 82.8 35 8.5	cross sectional Not identified online	3/ High risk of bias
Aldakhil, H. et al., 2021 [81]	2021.01 to 02	Saudi Arabia, EMR, Developing, High income	270 100 33 3	cross sectional Non-random sampling self-administration	6/ Low risk of bias
Kreuter, M.W. et al., 2022 [82]	2021.01	USA, AMR, Developed, High income	1951 96 26 2.5	cross sectional Non-random sampling online	5/ High risk of bias
Wan, X. et al., 2021 [83]	2020.12 to 2021.02	China, WPR, Developing, Upper intermediate income	468 68.38 30.5 4.5	cross sectional Random sampling self-administration	5/ High risk of bias
Wang, X. et al., 2021 [84]	2020.09 to 2021.04	China, WPR, Developing, Upper intermediate income	941 NR NR 1.4	cross sectional Non-random sampling face-to-face interview	5/ High risk of bias
Evans, S. et al., 2021 [85]	2021.01	Australia, WPR, Developed, High income	1094 83.1 39.2 8.9	cross sectional Non-random sampling online	5/ High risk of bias
Delgado-Gallegos, J.L. et al., 2021 [86]	2020.12 to 2021.02	México, AMR, Developing, Upper intermediate income	699 69.1 42 NR	cross sectional Non-random sampling online	7/ Low risk of bias
Yılmaz, M. et al., 2021 [87]	2021.02	Turkey, EUR, Developed, Upper intermediate income	1035 77.8 41.5 8.5	cross sectional Non-random sampling online	6/ Low risk of bias
Al-khlaiwi, T. et al., 2022 [88]	2021.01 to 03	Saudi Arabia, EMR, Developing, High income	1052 73.8 34 8.5	cross sectional Non-random sampling online	4/ High risk of bias
Derdemezis, C. et al., 2022 [89]	2020.10 to 2021.04	Greece, EUR, Developing, High income	1095 65.3 50.25 NR	cross sectional Non-random sampling online	6/ Low risk of bias
Yılmaz, M. et al., 2021 [87]	2021.02	Turkey, EUR, Developed, Upper intermediate income	1035 77.8 41.5 2	cross sectional Non-random sampling online	6/ Low risk of bias
Szilagyi, P.G. et al., 2021 [90]	2021.02 to 03	USA, AMR, Developed, High income	1745 57.91 34 9	cross sectional Non-random sampling online	5/ High risk of bias
Wang, Q. et al- wave 2, 2022 [67]	2021.02 to 03	China, WPR, Developing, Upper intermediate income	1038 67.3 41.5 NR	cross sectional Non-random sampling online	7/ Low risk of bias
Çağ, Y. et al., 2022 [91]	2021.03 to 04	Turkey, EUR, Developed, Upper intermediate income	1018 79.5 41.5 5.52	cross sectional Random sampling face to face interview	5/ High risk of bias
Teasdale, C.A. et al., 2021 [92]	2021.03 to 04	USA, AMR, Developed, High income	1119 59 41.5 6	cross sectional Random sampling self-administration	5/ High risk of bias
Teasdale, C.A. et al., 2021 [93]	2021.03	USA, AMR, Developed, High income	2074 49.5 41.5 6	cross sectional Random sampling self-administration	5/ High risk of bias
Schilling, S. et al., 2022 [94]	2021.02 to 03	USA, AMR, Developed, High income	50 98 32 9	cross sectional Non-random sampling face to face interview	6/ Low risk of bias
Skeens, M.A. et al., 2022 [95]	2021.02 to 05	USA, AMR, Developed, High income	491 89.5 38.79 9.16	cross sectional Non-random sampling online	6/ Low risk of bias
Alfieri, N.L. et al., 2021 [96]	2021.03	USA, AMR, Developed, High income	1425 NR NR 8.5	cross sectional Non-random sampling online	5/ High risk of bias
Lachance-Grzela, M. et al., 2022 [97]	2021.03 to 04	Canada, AMR, Developed, High income	406 NR NR 8.5	cross sectional Non-random sampling online	6/ Low risk of bias
Yoda, T. et al., 2021 [98]	2021.04	Japan, WPR, Developing, High income	1100 57.5 38.5 2.5	cross sectional Non-random sampling online	6/ Low risk of bias
Di Giuseppe, G. et al., 2022 [99]	2021.04 to 05	Italy, EUR, Developed, High income	607 82.4 42.5 9.5	cross sectional Random sampling online	6/ Low risk of bias
Bagateli, L.E. et al., 2021 [38]	2021.05 to 06	Brazil, AMR, Developing, Upper intermediate	501 85 34 8.5	cross sectional Non-random sampling online	5/ High risk of bias
Wang, Q. et al- wave 3, 2022 [67]	2021.05 to 06	China, WPR, Developing, Upper intermediate income	1183 57.5 41.5 NR	cross sectional Non-random sampling online	7/ Low risk of bias
Musa, S. et al., 2021 [100]	2021.05 to 06	Qatar, EMR, Developed, High income	4023 NR NR 13.5	cross sectional Non-random sampling online	5/ High risk of bias
Atad, E. et al., 2021 [101]	2021.04 to 05	Israel, EUR, Developed, High income	1118 NR NR 13.5	cross sectional Non-random sampling online	5/ High risk of bias
Al-Nafeesah, A.S. et al., 2021 [102]	2021.05	Saudi Arabia, EMR, Developing, High income	1143 88 41.5 3	cross sectional Non-random sampling online	5/ High risk of bias
Choi, S.-H. et al., 2021 [103]	2021.05 to 06	Korea, SEAR, Developed, High income	226 79.6 41.5 14	cross sectional Non-random sampling self-administration	5/ High risk of bias
Wagner, A. et al., 2022 [104]	2021.05	Switzerland, EUR, Developed, High income	1344 44.8 41 13.5	cross sectional Non-random sampling online	6/ Low risk of bias
Babicki, M. et al., 2021 [105]	2021.05	Poland, EUR, Developed, High income	4432 77.6 34 13.5	cross sectional Non-random sampling online	5/ High risk of bias
Horiuchi, S. et al., 2021 [106]	2021.05 to 06	Japan, WPR, Developing, High income	1200 49.1 34 8.5	cross sectional Not identified online	6/ Low risk of bias
Choi, K. et al., 2021 [107]	2021.05 to 07	USA, AMR, Developed, High income	322 NR NR 9.5	cross sectional Random sampling self-administration	5/ High risk of bias
Samannodi, M. et al., 2021 [108]	2021.06 to 07	Saudi Arabia, EMR, Developing, High income	508 61.3 39 9	cross sectional Non-random sampling online	6/ Low risk of bias
Gendler, Y. et al., 2021 [109]	2021.06	Israel, EUR, Developed, High income	520 77.1 44.76 13.5	cross sectional Non-random sampling online	6/ Low risk of bias
Zona, S. et al., 2021 [110]	2021.05 to 07	Italy, EUR, Developed, High income	1799 72.4 34 14.5	cross sectional Non-random sampling online	5/ High risk of bias
McKinnon, B. et al., 2021 [111]	2021.05 to 06	Canada, AMR, Developed, High income	809 NR NR 10	cohort Not identified online	5/ High risk of bias
Almusbah, Z. et al., 2021 [112]	2021.05 to 06	Saudi Arabia, EMR, Developing, High income	1000 47 NR 7	cross sectional Non-random sampling online	4/ High risk of bias
Urrunaga-Pastor, D. et al., 2021 [113]	2021.05 to 07	Latin American countries, AMR, Developed	227,740 38.37 36 NR	cross sectional Non-random sampling online	5/ High risk of bias
Kocamaz, E.B. et al., 2022 [114]	2021.05 to 06	Turkey, EUR, Developed, Upper-intermediate income	384 68.8 43 9	cross sectional Non-random sampling online	7/ Low risk of bias
Griva, K. et al., 2021 [115]	2021.06 to 07	Singapore, WPR, Developing, High income	1623 60.8 46.3 15	cohort Not identified face to face	5/ High risk of bias
Alhazza, S.F. et al., 2021 [116]	2021.06	Saudi Arabia, EMR, Developing, High income	1052 51.5 35 10	cross sectional Not identified online	7/ Low risk of bias
McElfish, P.A. et al., 2022 [117]	2021.07	USA, AMR, Developed, High income	189 53 41.5 14.5	cross sectional Random sampling phone interview	5/ High risk of bias
Russo, L. et al., 2021 [118]	2021.07 to 08	Italy, EUR, Developed, High income	1205 81.6 42 6	cross sectional Non-random sampling online	5/ High risk of bias
McElfish, P.A. et al., 2022 [117]	2021.07	USA, AMR, Developed, High income	168 48 41.5 5.5	cross sectional Random sampling phone interview	5/ High risk of bias
Temsah, M.H. et al., 2021 [119]	2021.07	India, SEAR, Developing, Low income	3167 65 41.5 15	cross sectional Non-random sampling online	6/ Low risk of bias
Mohan, R. et al., 2022 [120]	2021.07 to 09	India, SEAR, Developing, Low income	204 49.5 34 8.5	cross sectional Non-random sampling online	6/ Low risk of bias
Galanis, P. et al., 2021 [121]	2021.09	Greece, EUR, Developing, High income	813 76.1 42.3 14.5	cross sectional Non-random sampling online	5/ High risk of bias
Willis, D.E. et al., 2022, sample 1 [122]	2021.09 to 10	USA, AMR, Developed, High income	342 54.39 35 5.5	cross sectional Non-random sampling online	5/ High risk of bias
Shmueli, L., 2021 [123]	2021.09 to 10	Israel, EUR, Developed, High income	1012 NR NR 8	cross sectional Non-random sampling online	5/ High risk of bias
Willis, D.E. et al., 2022, sample 2 [122]	2021.09 to 10	USA, AMR, Developed, High income	321 51.09 35 14.5	cross sectional Non-random sampling online	5/ High risk of bias
Ma, L. et al., 2022 [124]	2021.09 to 10	China, WPR, Developing, Upper-intermediate income	9424 74.79 40 3	cross sectional Non-random sampling online	6/ Low risk of bias
Ali, M. et al., 2022 [125]	2021.10	Bangladesh, SEAR, Developing, Low income	2633 52.8 35 9	cross sectional Random sampling face-to-face interview	7/ Low risk of bias
Li, T. et al., 2022 [126]	2021.10 to 11	China, WPR, Developing, Upper-intermediate income	3342 64 41.5 10	cross sectional Non-random sampling online	6/ Low risk of bias
Ali, M. et al., 2022 [127]	2021.10	Bangladesh, SEAR, Developing, Low income	396 60.4 34.5 8.5	cross sectional Random sampling face-to-face interview	7/ Low risk of bias
Fisher, C.B. et al., 2021 [128]	2021.10	USA, AMR, Developed, High income	400 NR 35 7.4	cross sectional Non-random sampling online	4/ High risk of bias
Al-Qerem, W. et al., 2022 [129]	2021.09 to 11	Jordan, EMR, Developing, Upper-intermediate income	819 70.9 39 9	cross sectional Non-random sampling online	5/ High risk of bias
Tsai, C.S. et al., 2022 [130]	2021.08 to 2022.01	Taiwan, WPR, Developed, High income	252 NR 42.23 11.5	cross sectional Not identified self-administration	6/ Low risk of bias
Kheil, M.H. et al., 2022 [131]	2021.10 to 11	USA, AMR, Developed, High income	1746 55 53 NR	cross sectional Non-random sampling online	5/ High risk of bias
Al-Qerem, W. et al., 2022 [132]	2021.09 to 2022.02	Iraq, EMR, Developing, Upper intermediate income	491 59.3 29 9	cross sectional Non-random sampling online	7/ Low risk of bias
Almalki, O.S. et al., 2022 [133]	2021.11	Saudi Arabia, EMR, Developing, High income	4135 81 41.5 8	cross sectional Random sampling online	5/ High risk of bias
Miraglia del Giudice, G. et al., 2022 [134]	2021.12 to 2022.01	Italy, EUR, Developed, High income	427 86.5 41 8	cross sectional Random sampling phone interview	7/ Low risk of bias
Buonsenso et al., 2022 [135]	2021.11 to 2022.01	Italy, EUR, Developed, High income	121 80 42.5 7	cross sectional Non-random sampling phone interview	5/ High risk of bias
Miliordos, K. et al., 2022 [136]	2021.12 to 2022.01	Greece, EUR, Developing, High income	366 58.2 35 8	cross sectional Not identified face-to-face interview	5/ High risk of bias
Lau et al., 2022 [137]	2022.01	Hong Kong, WPR, Developed, High income	11,141 86 40 8.5	cross sectional Non-random sampling online	5/ High risk of bias
Aljamaan, F. et al., 2022 [138]	2022.01	Saudi Arabia, EMR, Developing, High income	1340 65.3 44.5 8	cross sectional Non-random sampling online	4/ High risk of bias
Aljamaan, F. et al., 2022 [138]	2022.01	Saudi Arabia, EMR, Developing, High income	1340 65.3 44.5 15	cross sectional Non-random sampling online	4/ High risk of bias
Altulaihi, B.A. et al., 2021 [139]	NR	Saudi Arabia, EMR, Developing, High income	333 NR 41.5 8.5	cross sectional Non-random sampling self-administration	3/ High risk of bias
Ruggiero, K.M. et al., 2021 [140]	NR	USA, AMR, Developed, High income	427 NR NR 9.5	cross sectional Non-random sampling online	7/ Low risk of bias
Al Yamani, Z.J. et al., 2022 [141]	NR	Saudi Arabia, EMR, Developing, High income	375 33.9 NR 3.5	cross sectional Random sampling self-administration	6/ Low risk of bias
Yigit, M. et al., 2021 [142]	NR	Turkey, EUR, Developed, Upper intermediate income	428 63.6 39.7 NR	cross sectional Non-random sampling face-to-face interview	5/ High risk of bias

**Table 2:** Subgroup analyses.

Subgroups		No. of Studies	ES (95% CI)	I^2^ (%)	Heterogeneity between Subgroups
Risk of bias	Low risk of bias	40	61 (56; 67)	99.76	0.13
	High Risk of bias	58	54 (46; 62)	99.94	
Country development status	Developed	53	55 (47; 63)	99.93	0.46
	Developing	45	59 (52; 66)	99.87	
Country Income level	Low income	4	62 (50; 73)	98.61	0.01
	Upper-intermediate income	29	65 (59; 71)	99.80	
	High income	62	52 (46; 58)	99.75	
	Multiple countries	3	76 (56; 96)	-	
Country location in WHO’s regions	Americas (AMRO)	27	58 (50; 67)	99.82	<0.001
	South-East Asia (SEARO)	5	62 (52; 72)	98.22	
	Europe (EURO)	24	51 (43; 59)	99.49	
	Eastern Mediterranean (EMRO)	17	45 (34; 56)	99.70	
	Western Pacific (WPRO)	23	67 (56; 78)	99.95	
	Multiple countries	2	69 (68; 70)	-	
Sampling method	Random sampling	15	52 (39; 65)	99.74	0.46
	Non-random sampling	74	59 (53; 64)	99.92	
	Not identified	9	50 (31; 69)	99.79	
Validated measure for assessing parents’ willingness to vaccinate their children	Yes	50	56 (50; 62)	99.90	0.13
	No	40	56 (48; 64)	99.87	
	Not identified	8	67 (57; 77)	99.52	
Data collection method	Online	72	58 (53; 64)	99.93	0.02
	Self-administered	13	56 (48; 63)	98.52	
	Phone interview	4	35 (22; 49)	94.42	
	Face-to-face Interview	9	55 (33; 76)	99.85	

**Table 3:** Results of uni-variable meta-regression regarding estimated pooled prevalence.

Variable	Number of Studies	Coefficient	S.E.	*p*	I^2^ res. (%)	Adj. R^2^ (%)	τ^2^
Percentage of mothers participated in study	83	0.001	0.002	0.51	99.87	−0.68	0.04
Mean age of parents	86	−0.004	0.005	0.46	99.90	−0.51	0.05
Mid-range of children’s age	82	−0.007	0.007	0.27	99.86	0.32	0.04
Data collection time	94	−0.14	0.04	0.001	99.92	9.78	0.04
Country % of people received at least one dose of COVID-19 vaccine at time of data collection	70	−0.001	0.001	0.11	99.83	2.39	0.04
Country % of people fully vaccinated with COVID-19 vaccine at time of data collection	70	−0.001	0.001	0.15	99.83	1.63	0.04

**Table 4:** Results of multivariable meta-regression.

Variable	Coefficient	S.E.	*p*	Model Summary
Data collection time	−0.13	0.04	0.003	Number of studies: 91 tau^2^: 0.04
Country income level	−0.07	0.04	0.06	I^2^ res. (%): 99.76
Country location in WHO’s regions	0.003	0.01	0.82	Adj. R^2^ (%): 11.44
Data collection method	0.007	0.03	0.81	

## Data Availability

Data will be available via email to corresponding author.
